# Auranofin induces apoptosis by ROS-mediated ER stress and mitochondrial dysfunction and displayed synergistic lethality with piperlongumine in gastric cancer

**DOI:** 10.18632/oncotarget.5364

**Published:** 2015-09-28

**Authors:** Peng Zou, Minxiao Chen, Jiansong Ji, Weiqian Chen, Xi Chen, Shilong Ying, Junru Zhang, Ziheng Zhang, Zhiguo Liu, Shulin Yang, Guang Liang

**Affiliations:** ^1^ Chemical Biology Research Center, School of Pharmaceutical Sciences, Wenzhou Medical University, Wenzhou, Zhejiang 325035, China; ^2^ School of Environmental and Biological Engineering, Nanjing University of Science and Technology, Nanjing, Jiangsu 210094, China; ^3^ Department of Pharmacy, The First Affiliated Hospital of Wenzhou Medical University, Wenzhou, Zhejiang 325035, China; ^4^ Department of Interventional Radiology, The Fifth Affiliated Hospital of Wenzhou Medical University, Lishui, Zhejiang 323000, China

**Keywords:** ROS, auranofin, piperlongumine, ER stress, mitochondrial dysfunction

## Abstract

Gastric cancer (GC) is one of the leading causes of cancer mortality in the world. In addressing the need of treatments for relapsed disease, we report the identification of an existing U.S. Food and Drug Administration-approved small-molecule drug to repurpose for GC treatment. Auranofin (AF), clinically used to treat rheumatic arthritis, but it exhibited preclinical efficacy in GC cells. By increasing intracellular reactive oxygen species (ROS) levels, AF induces a lethal endoplasmic reticulum stress response and mitochondrial dysfunction in cultured GC cells. Blockage of ROS production reversed AF-induced ER stress and mitochondrial pathways activation as well as apoptosis. In addition, AF displays synergistic lethality with an ROS-generating agent piperlongumine, which is a natural product isolated from the long pepper *Piper longum* L. Taken together, this work provides a novel anticancer candidate for the treatment of gastric cancer. More importantly, it reveals that increased ROS generation might be an effective strategy in treating human gastric cancer.

## INTRODUCTION

Gastric cancer (GC) is the second leading cause of cancer-related deaths in the world and is clinically challenging, especially in East Asia [1]. Current treatment modalities for GC include surgery, radiotherapy, chemotherapy, and their combinations. Although surgery is the main treatment for curing gastric cancer, adjuvant or perioperative chemotherapy and molecule-targeted chemotherapy have been prescribed for gastric cancer, due to their marked benefits in reducing disease recurrence and increasing long-term survival [2, 3]. However, severe side effects and complications such as hematological and gastrointestinal toxicities of current anticancer drugs become major problems in the clinical setting, which highlights the urgent need for novel effective and less toxic therapeutic approaches [4, 5]. Therefore, novel effective and safe treatments need to be developed and tested. Thus, repurposing of an existing U.S. Food and Drug Administration (FDA)-approved small-molecule drug in the treatment of gastric cancer is a worthy goal [6].

Compared with normal cells, cancer cells have intrinsically higher levels of reactive oxygen species (ROS) and are under oxidative stress due to an imbalanced redox status [7]. Elevated ROS levels also render cancer cells more sensitive to agents that further increase ROS and oxidative stress [8, 9]. Auranofin (AF) is a metal phosphine complex that has been used for the clinical treatment of rheumatoid arthritis, following the pioneering studies conducted with gold (I) thiolate compounds [10]. Previous studies suggested that AF acted as an inhibitor of thioredoxin reductase, which could cause the oxidative damage and modifications of cellular redox status, resulting in over production of ROS and apoptosis [11, 12]. AF can exert a strong cytotoxic effect on several different types of neoplastic cells both *in vitro* and *in vivo* [13, 14]. The cytotoxic activity of AF along with its relative safe profile in patients warrants the application potential of AF in cancer therapy and other diseases [15]. AF is currently in clinical trials for the treatment of leukemia, recurrent ovarian epithelial cancer and recurrent non-small cell lung carcinoma (https://clinicaltrials.gov/ct2/show/NCT01419691, NCT01747798, NCT01737502). Although it has been tested in several types of human malignancies, AF has not yet been tested in GC.

In this study, we showed that AF could induce GC cells apoptosis via activating ROS-dependent ER stress and mitochondrial pathways, blockage of ROS production by specific inhibitor totally abolished the anti-cancer effects of AF. Moreover, we evaluated the synergistic inhibitory effect of AF in GC cells with Piperlongumin (PL), which is a natural product isolated from the long pepper *Piper longum* L that was recently identified as selectively toxic to cancer cells in ROS-dependent manner [16, 17]. The mechanistic investigation elucidated that PL as a ROS inducer could dramatically enhance AF-induced GC cells apoptosis *in vitro* and *in vivo* by triggering ROS-mediated ER stress pathway. Our study indicates that ROS production could be an important target for developing new anti-cancer drugs.

## RESULTS

### AF causes loss of viability of human gastric cancer cells

**Figure 1:**
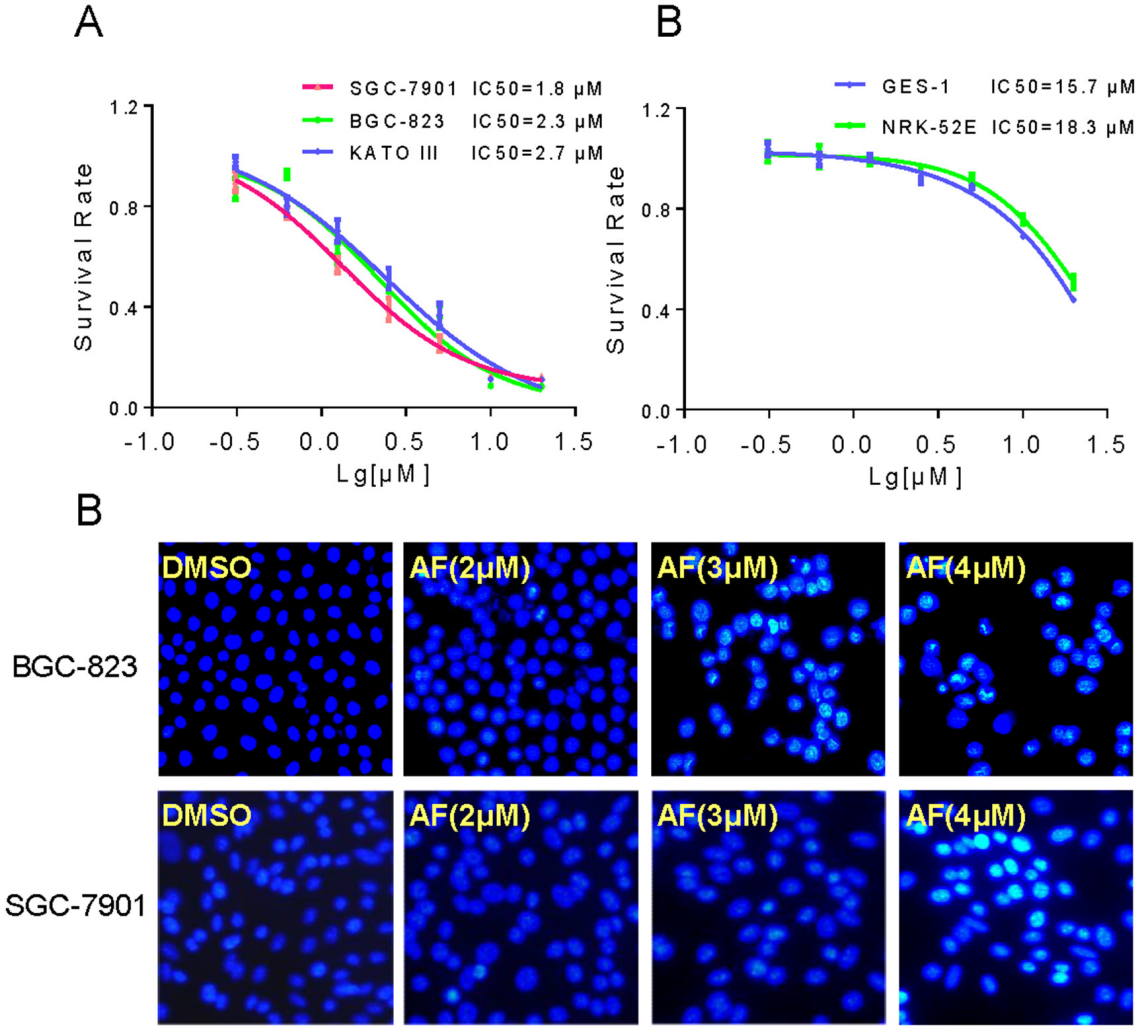
AF inhibits gastric cancer cells growth **A–B.** The effects of AF on the proliferation of human gastric cancer cells and normal cells. BGC-823, SGC-7901, KATO III, GES-1 or NRK-52E cells were incubated with increasing doses of AF (0.625–20 μM) for 24 h respectively. Cell viability was determined by MTT assay and the IC_50_ values were calculated. **C.** AF treatment induced apoptotic morphology in BGC-823 and SGC-7901 cells. BGC-823 or SGC-7901 cells were treated with AF (2, 3 or 4 μM) for 12 h. Cell morphology was observed using an inverted microscope after Hoechst 33258 staining.

To investigate the effect of AF on the growth of human gastric cancer cells, BGC-823, SGC-7901 and KATO III cells were treated with AF *in vitro* for 24 hours and cell viability was detected by the MTT assay. As shown in Figure 1A, AF dose-dependently decreased the cell viability in BGC-823, SGC-7901 and KATO III cells with IC_50_ values of 2.3, 1.8 and 2.7 μM, respectively. By contrast, only a small percentage of cell death was found in normal cells after treated with 5 μM AF for 24 h. However, at the highest dose of 20 μM, AF treatment suppressed the growth of normal cells (Figure 1B). Further, morphological changes were determined using Hoechst 33258 staining in BGC-823 and SGC-7901 cells. Figure 1C revealed that treatment with AF resulted in a dose-dependent increase in the number of apoptotic cells, and exhibited significant apoptotic characteristics such as nuclear condensation and fragmentation.

### AF induced apoptosis in human gastric cancer cells

**Figure 2:**
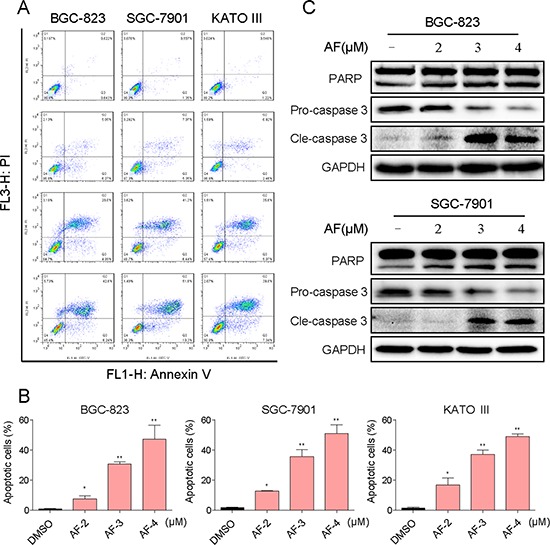
AF induces apoptosis in human gastric cancer cells **A.** Induction of apoptosis in human gastric cancer cells was determined by flow cytometry after treatment with AF (2, 3 or 4 μM) for 24 h. Similar results were obtained in three independent experiments. **B.** The percentage of apoptotic cells in the treatment groups was calculated (*p < 0.05, **p < 0.01). **C.** BGC-823 or SGC-7901 cells were treated with AF (2, 3 or 4 μM) for 20 h. Whole-cell lysates were subjected to western blot to assess the expression of cell apoptosis related proteins. GAPDH was used as internal control. Data represent similar results from three independent experiments.

We further examined the pro-apoptosis effect of AF on human gastric cancer cells using Annexin V/Propidium Iodide (PI) staining assay. As shown in Figure 2A and 2B, all of three gastric cancer cell lines have shown a concentration-dependent apoptosis after a 24 h treatment with AF. Then we determined the levels of apoptosis-related proteins in BGC-823 and SGC-7901 cells treated with AF. Figure 2C showed that treatment with AF for 20 h dose-dependently activated caspase-3/PARP pathway and increased the level of cleaved caspase-3/PARP, suggesting that AF-induced apoptosis may be associated to caspase-3/PARP pathway activation.

### ROS generation is the regulator of AF-induced apoptosis

**Figure 3:**
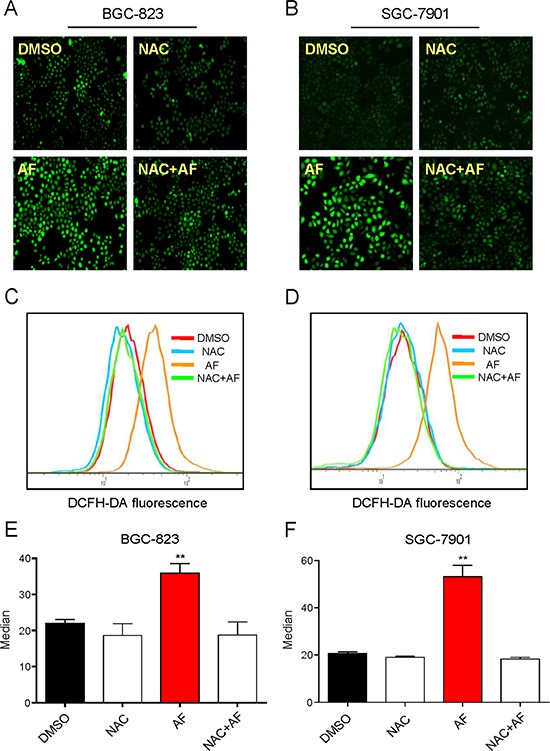
Treatment with AF induces ROS accumulation in human gastric cancer cells **A–B.** Intracellular ROS generation induced by AF was measured in BGC-823 A. or SGC-7901 B. cells by staining with DCFH-DA (10 μM) for 0.5 h. Fluorescence images were acquired by fluorescence microscopy. **C–D.** Intracellular ROS levels were measured by flow cytometry. **E–F.** Flow cytometry results from (C and D) were calculated and represented as the percent of control (*p < 0.05, **p < 0.01). A-F. NAC completely blocked ROS generation induced by AF. Cells were pretreated with or without 5 mM NAC for 2 h before exposure to AF for 2 h, intracellular ROS levels were measured by fluorescence microscopy A–B. or flow cytometry C–D. Data presented are representative of three independent experiments.

**Figure 4:**
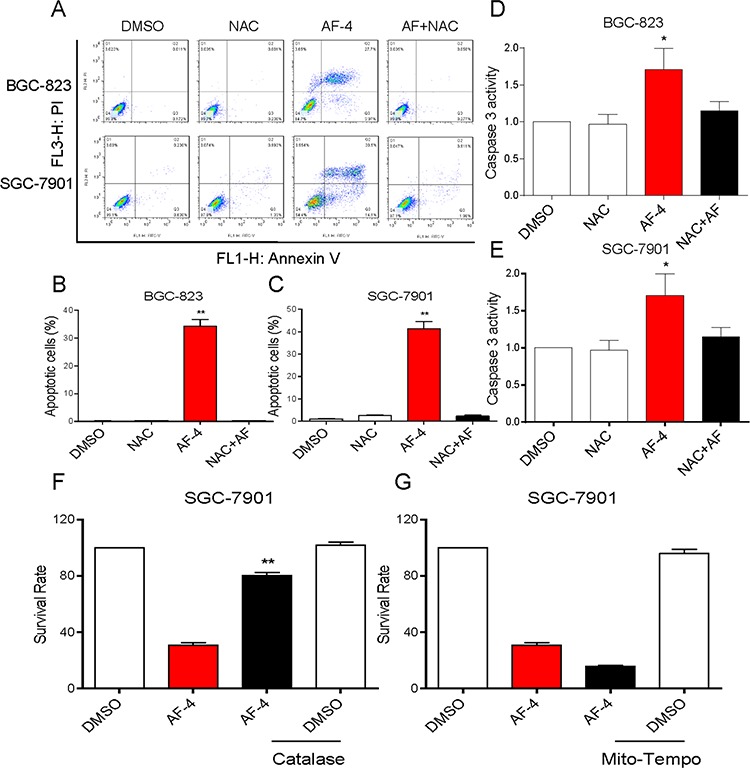
AF induces cytotoxicity in human gastric cancer cells is dependent on intracellular ROS generation **A.** Blocking of ROS generation abolished the cytotoxicity of AF. BGC-823 or SGC-7901 Cells were pre-incubated with or without 5 mM NAC for 2 h before exposure to AF (4 μM) for 24 h. Percentage of cell apoptosis was determined by Annexin-V/PI staining and flow cytometry. **B–C.** The percentage of apoptotic cells in the treatment groups was calculated. Assays were performed in triplicate. **D–E.** NAC totally reversed the activation of caspase-3 caused by AF in gastric cancer cells. Cells were pre-incubated with or without 5 mM NAC for 2 h before exposure to AF (4 μM) for 20 h and the caspase-3 activity in the cell extracts was determined by a assay kit using specific substrate. **F.** Reversion of AF-induced death by catalase. SGC-7901 cells were pretreated with catalase (CAT, 2,000 U/mL) for 2 h, followed by 4 μM AF for 24 h. Cell viability was determined by MTT assay. **G.** AF-induced cell death is not reversed by Mito-Tempo. SGC-7901 cells were pretreated with Mito-Tempo (10 μM) for 2 h, followed by 4 μM AF for 24 h. Cell viability was determined by MTT assay. (*p < 0.05, **p < 0.01).

It was believed that AF could induce ROS-mediated apoptosis by inhibiting TrxR1 activity [11]. Therefore, we determined the roles of ROS in AF-induced cytotoxicity in human gastric cancer cells. As shown in Figure 3A–3F, treatment with AF for 2 h in BGC-823 and SGC-7901 cells caused a significantly increase in DCF-reactive ROS. Pretreatment with the antioxidant N-acetyl cysteine (NAC, 5 mM) completely inhibited AF-mediated ROS induction. In addition, we found that NAC almost completely abolished BGC-823 and SGC-7901 cells apoptosis induced by AF (Figure 4A–4C). Similar results were observed in the caspase-3 activity assay (Figure 4D–4E). We also found that pretreatment with catalase (2000 U/mL) for 2 h significantly abolished SGC-7901 cells death induced by AF (Figure 4F). However, pretreatment with Mito-Tempo (a mitochondria-specific antioxidant) for 2 h failed to protect cells against AF-induced cell death, suggesting that mitochondrial ROS was not involved in the event of AF-mediated cell death (Figure 4G).

### AF-induced ROS increases ER stress and mitochondrial dysfunction, which contributes to AF lethality in gastric cancer cells

**Figure 5:**
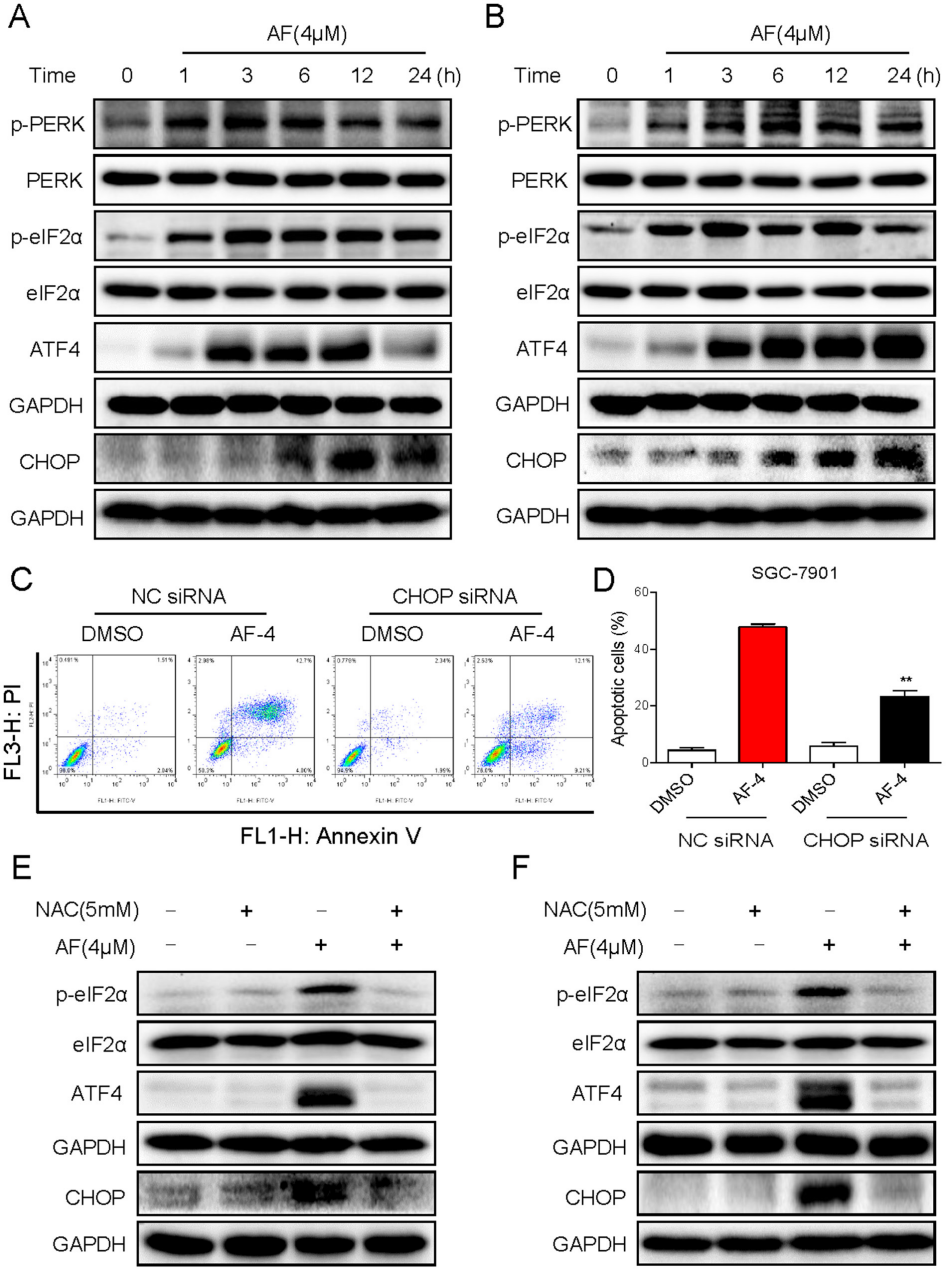
Treatment with AF induces ER stress in human gastric cancer cells **A–B.** BGC-823 **A.** or SGC-7901 **B.** cells were treated with AF (4 μM) for the indicated times, the protein levels of p-PERK, p-eIF2α, ATF4 and CHOP were determined by western blot. **C–D.** SGC-7901 cells transfected with CHOP siRNA or control siRNA were treated with AF (4 μM) for 24 h. Percentage of cell apoptosis was determined by Annexin-V/PI staining and flow cytometry. **E–F.** NAC totally reversed the activation of ER stress induced by AF. BGC-823 or SGC-7901 cells were pretreated with or without 5 mM NAC for 2 h before exposure to AF, three hours later the p-eIF2α and ATF-4 expression were detected by western blot. The protein level of CHOP was examined by western blot after treatment with AF for 12 h. GAPDH was used as internal control. Data presented are representative of three independent experiments.

Increased ROS levels and perturbation in the intracellular redox status increase the levels of unfolded proteins in the ER and induce ER stress response [18, 19]. UPR induces PERK-mediated phosphorylation of eukaryotic initiation factor-2α, which blocks cap-dependent protein translation but allows preferential translation of ATF4. While upregulating chaperone proteins, for example, GRP78, required in restoring the ER function, ATF4 also induces the prodeath transcriptional regulator CHOP [20, 21]. Thus, we next examined the expressions of ER stress-related proteins, such as p-PERK, p-eIF2α, ATF4, and CHOP in AF-treated gastric cancer cells. The time-course result indicated that AF (4 μM) could significantly activate ER stress. The expression levels of p-PERK, p-eIF2α and ATF4 reached the peak at 3–6 h after treatment, and CHOP peaked after 12 h treatment in BGC-823 cells (Figure 5A). Similar results were observed in SGC-7901 cells (Figure 5B). In order to further confirm that ER stress plays an important role in the induction of gastric cancer cells apoptosis by AF, CHOP expression was downregulated by CHOP siRNA. As shown in Figure 5C–5D, knockdown of CHOP by siRNA, markedly attenuated AF-induced apoptosis in SGC-7901 cells. In addition, we found that pretreatment with the antioxidant NAC completely blocked the expression of p-eIF2α, ATF4 and CHOP in AF-treated BGC-823 and SGC-7901 cells (Figure 5E and 5F), and totally inhibited apoptosis of gastric cancer cells, as shown above in Figure 4A. These findings demonstrate that AF-induced ROS could leads to induction of ER stress, associated with increase in the expression of p-PERK, p-eIF2α, ATF4 and CHOP in gastric cancer cells.

**Figure 6:**
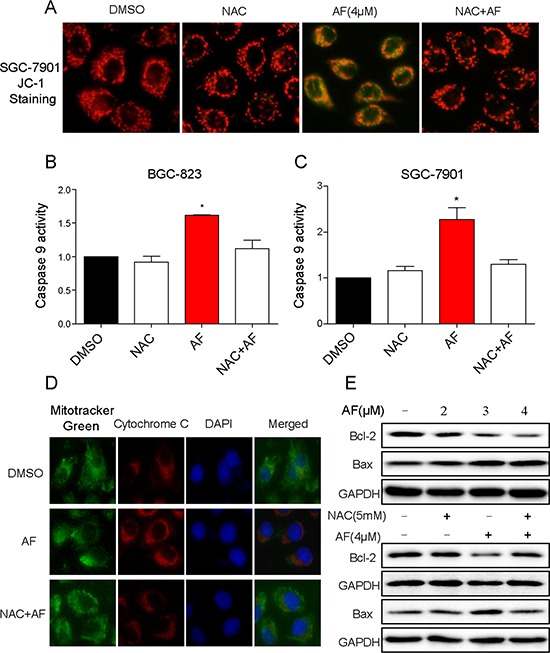
AF activates mitochondrial apoptotic pathway **A.** Treatment of cells with AF obviously decreased the mitochondrial membrane potential (Δ*ψ*_m_). SGC-7901 cells after treatment AF (4 μM) for 14 h were stained with JC-1 and then analyzed by fluorescence microscope. **B–C.** Caspase-9 activity. Cells after treatment with AF for 20 h were lysed and cell proteins were used to determine caspase-9 activity with assay kit using specific substrate. **D.** Treatment with AF provoked the release of cytochrome C from mitochondria into cytosol. SGC-7901 cells after treatment AF (4 μM) for 14 h were stained with Mito-T green fluorescence, anti-cytochrome C and DAPI for detecting the translocation of cytochrome C from the mitochondria to the cytosol. **E.** Western blot analysis effects of AF on expression of Bcl-2 family proteins in SGC-7901 cells. A–E. NAC significantly reversed the activation of mitochondrial apoptotic pathway induced by AF. All images shown here are representative of three independent experiments with similar results.

It is well known that mitochondria are central to the regulation of apoptosis [22]. Excessive generation of ROS renders the cells oxidatively stressed and impairs membrane proteins, leading to mitochondrial dysfunction. Loss of mitochondrial membrane potential (Δ*ψ*_m_) is catastrophic for cells and leads to the release of cytochrome C into the cytosol [23]. Therefore, fluorescence microscope analysis was used to confirm whether AF treatment-induced apoptosis occurred through destroying mitochondrial homeostasis using JC-1 as a molecular probe. As shown in Figure 6A, the integrity of mitochondrial membranes potential was decreased in SGC-7901 cells after AF treatment as evidenced by the elevation of green fluorescence from the red to green. The release of cytochrome C to the cytoplasm and caspase 9 activity were both elevated in SGC-7901 cells (Figure 6B–6D). The imbalance of pro-apoptotic and anti-apoptotic Bcl-2 family proteins would lead to the loss of mitochondrial membrane potential and finally result in the induction of apoptosis. Hence, it was of interest to identify the Bcl-2 family members involved in the AF treatment-induced apoptosis. As shown in Figure 6E, treatment with AF remarkably decreased the expression of Bcl-2, bur increased the expression of Bax in SGC-7901 cells. In addition, pre-incubation with NAC attenuated these effects confirming their linkage to AF-induced oxidative stress (Figure 6A–6E). Collectively these results indicated that AF treatment induced ROS-dependent mitochondrial apoptosis in gastric cancer cells.

### PL enhances AF-induced cell apoptosis by elevating intracellular ROS levels and ER stress

**Figure 7:**
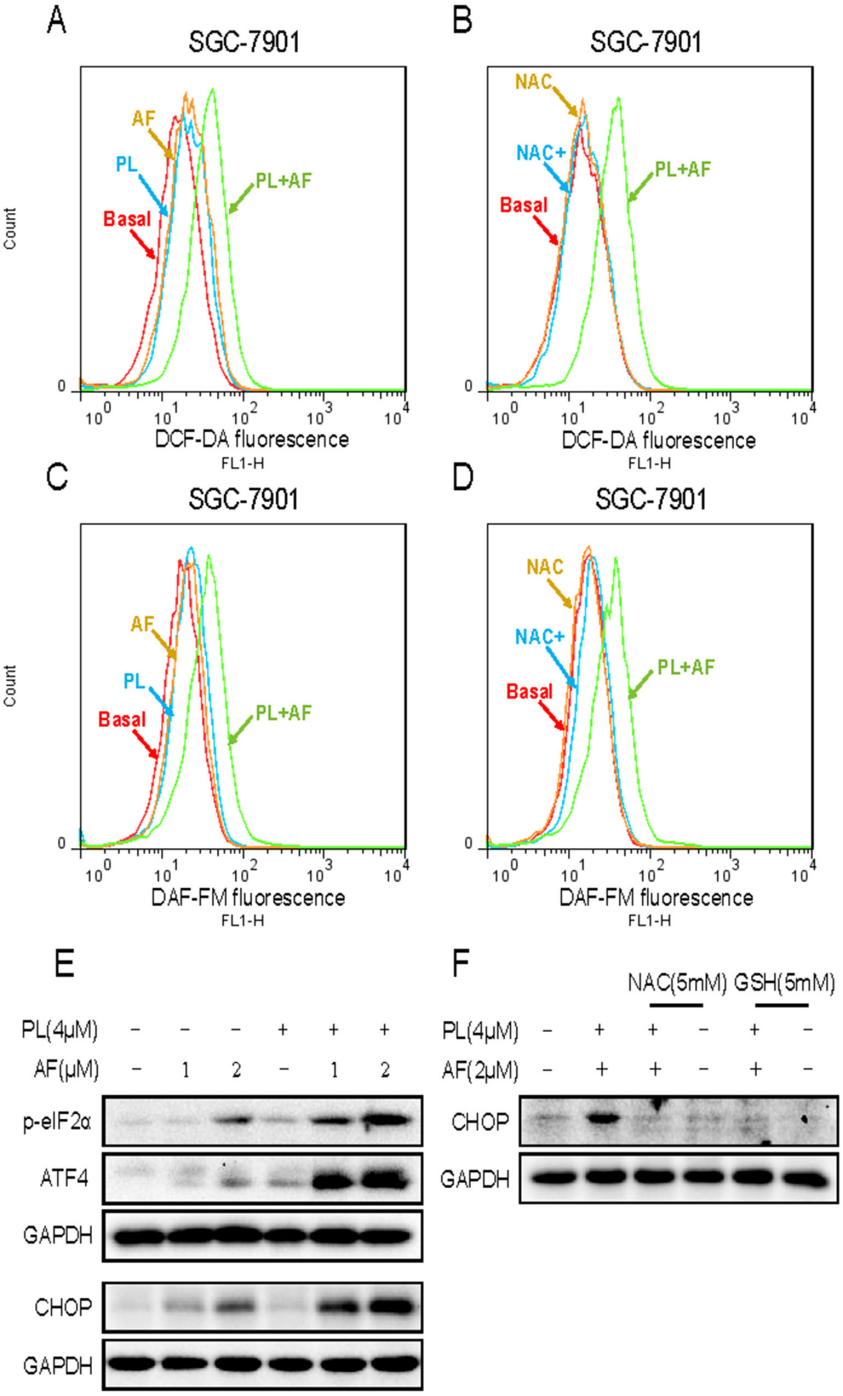
PL enhances AF-induced intracellular ROS accumulation and ER stress response **A** and **C.** PL enhances AF-induced intracellular ROS accumulation. Intracellular ROS levels were measured in SGC-7901 cells by staining with DCFH-DA (10 μM) or 4-amino-5-methylamino-2′,7′-difluorofluorescein (DAF-FM) diacetate (5 μM) and flow cytometry analysis. **B** and **D.** NAC blocked combined treatment-induced intracellular ROS accumulation. SGC-7901 cells were pre-incubated with 5 mM NAC for 2 h before combined treatment for 2 h. Intracellular ROS levels were measured by flow cytometry. **E.** PL enhances AF-induced ER stress response. SGC-7901 cells after treatment were collected and lysed, the protein levels of p-eIF2α, ATF4 and CHOP were determined by western blot. GAPDH was used as internal control. **F.** NAC or GSH addition reversed combined treatment-induced ER stress response. All images shown here are representative of three independent experiments with similar results.

PL is reported to produce ROS as a major of its anticancer mechanisms [16, 17]. Our research found that AF could induce gastric cancer cells apoptosis through ROS-mediated oxidative damage. Hence, we asked whether PL could enhance the ROS accumulation induced by AF. As shown in Figure 7A, treatment of cell with AF (2 μM) and PL (4 μM) alone both slightly induced ROS accumulation, but combined treatment with PL and AF resulted in significant increases in ROS level. Using a fluorescent probe specific for individual species of ROS, we found that nitric oxide also induced by combined treatment with PL and AF in SGC-7901 cells (Figure 7B). Pretreatment with NAC fully reversed the increases in the levels of ROS and nitric oxide induced by combined treatment (Figure 7C and 7D). Abundant intracellular ROS may cause DNA damage and activate down-streamed signaling pathway. Therefore, it was interested to investigate whether the ER-stress pathway was activated by combined treatment. As shown in Figure 7E, treatment of cell with AF and PL alone both slightly induced the expressions of p-eIF2α and ATF4. However, AF and PL in combination dramatically activated ER-stress pathway, as convinced by significantly enhanced expressions of p-eIF2α, ATF4, and CHOP. In additon, NAC pretreatment completely blocked the combined treatment-induced overexpression of CHOP in SGC-7901 cells (Figure 7F).

**Figure 8:**
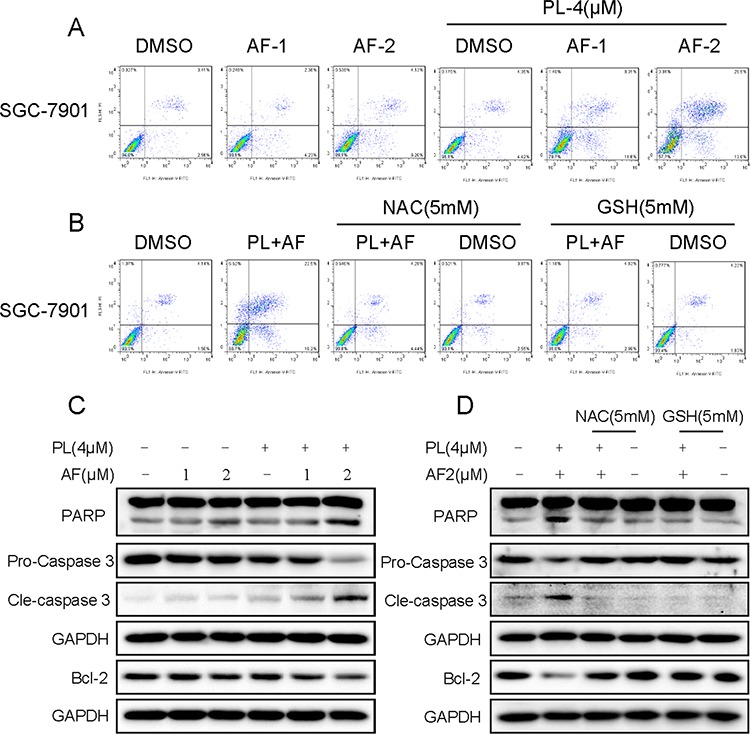
PL enhances AF-induced apoptosis in SGC-7901 cells **A.** PL enhances AF-induced apoptosis in SGC-7901 cells. Cells after treatment were collected and stained Annexin-V/PI, percentage of cell apoptosis was determined by flow cytometry, and ROS scavengers prevent SGC-7901 cells from combined treatment-induced cell apoptosis **B. C.** PL enhances AF-induced expression of cell apoptosis related proteins. Cells after treatment for 20 h were lysed, and the expression of apoptosis related proteins were detected by western blot. **D.** NAC or GSH addition reversed combined treatment-induced expression of apoptosis related proteins. Data presented are representative of three independent experiments.

To determine the combined effects of PL and AF, Annexin V/PI double staining detected by flow cytometry was employed to evaluate combined treatment-induced cell apoptosis. As shown in Figure 8A, compared with AF alone, cells treated with PL combination showed significant enhancement in apoptosis. This finding was further confirmed by cleavage of caspase-3 and PARP. As shown in Figure 8C, combination of AF and PL resulted in increased activation of caspase-3, together with increased cleavage of PARP in SGC-7901 cancer cells. In addition, AF and PL cotreatment significantly suppressed the expression level of anti-apoptotic protein Bcl-2 (Figure 8C). To confirm whether ROS accumulation is a necessary event in the potentiated apoptosis, two ROS scavenger, GSH and NAC were induced in our experiment. The results revealed that scavenging of ROS nearly reversed all the detected effects induced by the combined treatment, including the cleaving of caspase-3 and PARP (Figure 8B and 8D). Inhibition of Bcl-2 was also reversed (Figure 8D). These results revealed the vital role of ROS in the synergism. The redox system might be the upstream target for PL to enhance the apoptosis induced by AF in gastric cancer cells.

### PL amplifies the therapeutic effect of AF *in vivo*

**Figure 9:**
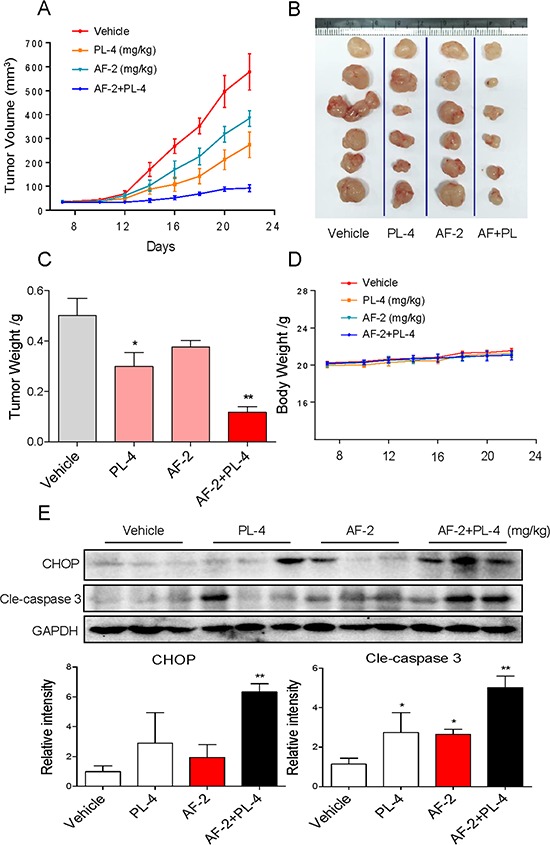
PL enhances AF-induced growth inhibition of tumor xenografts Combined treatment inhibits tumor volume **A** and **B.** and tumor weight **C.** of SGC-7901 human gastric cancer xenografts in nude mice, but do not affect body weight **D.** of mice. **E.** Western blot analysis on the expression of CHOP and caspase3 cleavage from respective tumor tissue lysates. GAPDH was used as protein loading control. Protein levels were quantified by Image-Pro Plus 6.0. (*p < 0.05, **p < 0.01).

To evaluate the synergetic effect of AF and PL *in vivo*, immuno-deficient nude mice bearing SGC-7901 tumor xenografts were employed to investigate the therapeutic effect of AF in combination with PL. As expected, after 14 days' administration, treatment of AF and PL alone both inhibited SGC-7901 tumor xenografts growth. However, SGC-7901 tumor xenografts growth in nude mice was more effectively inhibited by the combined treatment with AF and PL. For instance, combined treatment with AF and PL significantly inhibited the tumor volume (Figure 9A and 9B) and tumor weight (Figure 9C), but not affected body weight of mice (Figure 9D). The *in vivo* mechanistic studies revealed that combined treatment inhibited tumor xenografts by induction of caspase activation and CHOP expression (Figure 9E), which were consistent with the results *in vitro*. Taken together, these results all indicated that PL can synergizes the therapeutic effect of AF *in vivo*.

## DISCUSSION

Drug ‘repurposing’ is the identification of new therapeutic applications for drugs that have received US FDA approval for another purpose. Due to the reduced length and cost of research and trial phases, drug repurposing is more affordable and achievable than novel drug discovery [24]. Auranofin is a drug that is approved for the treatment of rheumatoid arthritis. Recently, treatment with AF was discovered to inhibit TrxR and induce ROS in cancer cells, which was associated with high *in vitro* and *in vivo* potency of AF against cancer cells [11, 25]. With the goal of potential repurposing AF for the treatment of gastric cancer, our preclinical studies presented here demonstrate that AF could induce ROS in the gastric cancer cells. Here, we also showed for the first time that treatment with AF was linked to and could induce lethal oxidative, ER stress-based UPR responses and mitochondrial dysfunction in gastric cancer cells. Furthermore, Co-treatment with NAC fully reversed the AF-induced increase in ROS and cell death, served to reinforce the role of ROS as an effector.

ROS plays a crucial role in the processes of tumor genesis, progression, and metastasis. Cancer cells usually possess higher levels of ROS and higher antioxidant activities in an uncontrolled status as compared to normal cells [26]. As a result, cancer cells are unable to cope with additional oxidative stress and become vulnerable to ROS [7, 27]. Therefore, targeting ROS is an important therapeutic strategy for cancer as exemplified by cancer drugs such as daunorubicin [28], cisplatin [29], paclitaxel [30], and trisenox [31]. Exploring the mechanisms of ROS based treatment is required for further improving the efficacy and specificity of cancer drugs.

In response to oxidative stress mediated by ROS, accumulation of unfolded or misfolded proteins triggers a cellular adaptive procedure known as ER stress [20]. Normally, ER stress is designed to be protective by mediating the shutdown of general protein synthesis and by increasing the production of molecular chaperones, including the ER resident hsp70 homologue, glucose-regulated protein 78 (GRP78) [21]. However, if ER stress is protracted, lethal ER stress ensues through prolonged activation of the pro-death ER stress pathways mediated by CHOP (CAAT/enhancer binding protein homologous protein) [32]. Our findings showed p-PERK, p-eIF2α and ATF4 were induced in a time-dependent manner after AF stimulation, suggesting that ER stress is activated at 3–6 h after AF treatment. These initiations of ER stress apoptotic pathway have been reported to increase CHOP gene expression, triggering ER stress-specific cascade for implementation of apoptosis. Consequently, the up-regulation of CHOP protein expression was observed in BGC-823 and SGC-7901 cells after AF treatment for 6–12 h. Furthermore, we found that AF-induced activation of ER stress was almost completely blocked by NAC, indicating that ROS production is the upstream regulator of AF-induced ER stress in gastric cancer cells.

Recently, combined (rather than single-agent) chemotherapy has been found to be a superior treatment strategy [33]. Hence, searching of effective chemosensitizers that could augment the efficiency of anticancer drugs and simultaneously overcome multi-drug resistance and side effects is urgently needed [34, 35]. Piperlongumine (PL) is a naturally occurring small molecule recently identified to be toxic selectively to cancer cells *in vitro* and *in vivo* [16]. This compound was found to elevate cellular levels of reactive oxygen species selectively in cancer cell lines [16, 36]. Therefore, we assessed the synergistic effects of PL and AF. The present study to show that PL potentiates the cytotoxic effect of AF in gastric cancer cells *in vitro* and *in vivo*. PL induced a robust increase in AF-mediated apoptosis via ROS-mediated ER-stress activation. To further characterize the importance of ROS in combined treatment, two antioxidants, GSH and NAC were employed. As anticipated, addition of GSH and NAC completely attenuated combined treatment-induced cell growth inhibition against SGC-7901 cells. Furthermore, both reduced PARP and caspase-3 cleavage detected by western blotting confirmed this protective effect of NAC and GSH. Therefore, based on these results, we proposed that PL enhanced AF-induced gastric cancer cell apoptosis by ROS overproduction.

The present study demonstrates that PL can act as a ROS inducer agent to enhance AF-induced human gastric cancer cell killing and apoptosis through ROS-mediated ER-stress and mitochondrial dysfunction pathways. It is reported that AF could bind to the SeC-containing C-terminal and the N-terminal redox center to inhibit TrxR activity [37], and PL probably directly binds to and inhibits the antioxidant enzyme glutathione S-transferase pi 1 (GSTP1) and carbonyl reductase 1 (CBR1), resulting in elevated levels of ROS and subsequent cancer-selective cell death [16, 17]. We speculate the possibility that PL inhibits GSTP1 or CBR1 activity and caused ROS accumulation, which in turn oxidized intracellular thiol-containing antioxidant agents like GSH and Trx, thus sensitized the cancer cells to AF-induced apoptosis. Further studies are necessary to comfirm the hypothesis. This finding predicts that PL has promising implications in improving the therapeutic efficacy when combining with other anticancer drugs in clinic.

**Figure 10:**
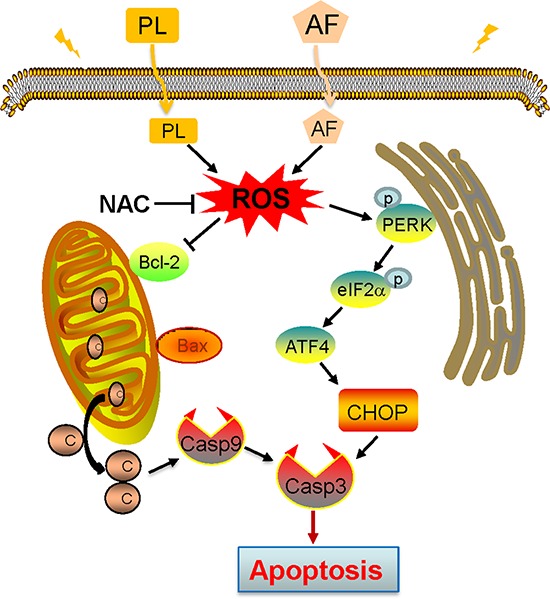
Proposed signal pathway. PL enhances AF-induced intracellular ROS accumulation ROS overproduction causes ER stress and mitochondria dysfunction, which in turn triggers the apoptotic signals.

In summary, we here investigated the anti-proliferative effects and mechanisms of AF, a FDA-approved small-molecule drug, in gastric cancer cell lines. We found that AF treatment resulted in severe ROS accumulation, excessive ROS caused the activation of ER stress and mitochondrial apoptotic pathways. We also demonstrated the synergistic effect of PL/AF combination on suppression of tumor growth *in vivo* using a xenograft tumor model. The suppression of apoptosis by NAC validates the critical role of ROS in combined treatment-induced cell death. Figure 10 showed a proposed signaling model leading to development of ROS-induced cell death induced by AF and PL. Our results suggested that combining low dose of PL with AF can serve as a potential combination therapy for the treatment of human gastric cancer. In addition, we also demonstrated that ROS production could be an important target for the development of new anti-cancer drugs.

## MATERIALS AND METHODS

### Cell culture and reagents

Auranofin (Santa Cruz, CA) and Piperlongumine (Sigma, St. Louis, MO) were suspended in dimethyl sulfoxide and stored in volumes of 1 mL at −20°C. Human gastric cancer cell lines SGC-7901, BGC-823 and KATO III were purchased from the Institute of Biochemistry and Cell Biology, Chinese Academy of Sciences. The cells were routinely cultured in RPMI 1640 medium (Gibco, Eggenstein, Germany) containing 10% heat-inactivated fetal bovine serum (Gibco, Eggenstein, Germany), 100 units/mL penicillin, and 100 μg/mL streptomycin in a humidified cell incubator with an atmosphere of 5% CO_2_ at 37°C. Antibodies including anti-p-PERK, anti-Bcl-2, anti-Bax, anti-cleaved PARP, anti-caspase-3 p30/17, anti-GAPDH, goat anti-mouse IgG-HRP and donkey anti-rabbit IgG-HRP were purchased from Santa Cruz Biotechnology (Santa Cruz, CA). Antibodies including anti-CHOP, anti-ATF4, anti-p-eIF2α, anti-Cleaved caspase-3 were purchased from Cell Signaling Technology (Danvers, MA). FITC Annexin V apoptosis Detection Kit I and Propidium Iodide (PI) were purchased from BD Pharmingen (Franklin Lakes, NJ).

### Cell viability assay

Cells were seeded into 96-well plates at a density of 8 × 10^3^ per well and allowed to grow overnight in RPMI 1640 containing 10% heat-inactivated FBS. AF was dissolved in DMSO and diluted with 1640 medium to final concentrations of 0.625, 1.25, 2.5, 5, 10, and 20 μM. The tumor cells were incubated with AF for 24 h before the MTT assay.

### Hoechst 33258 staining

At 12 h after AF (2, 3 or 4 μM) treatment, cells were fixed, washed twice with PBS and stained with Hoechst 33258 staining solution according to the manufacturer's instructions (Hoechst Staining Kit, Beyotime Biotechnology, China). Apoptotic features of cell death were determined by the staining of cell nuclei with the DNA-binding fluorochrome H33258 assessing chromatin condensation by using fluorescence microscope (Nikon, Japan) with 20X amplification. In each group, five microscopic fields were selected randomly.

### Measurement of reactive oxygen species generation

Cellular ROS contents were measured by flow cytometry as described previously [19]. Briefly, 5 × 10^5^ cells were plated on 60-mm dishes, allowed to attach overnight, and exposed to AF for 2 h. Cells were stained with 10 μM DCFH-DA or 5 μM DAF-FM-DA (Beyotime Biotech, Nantong, China) at 37°C for 30 min. Cells were collected and the fluorescence was analyzed using a FACSCalibur flow cytometer (BD Biosciences, CA). In some experiments, cells were pretreated with 5 mM NAC or 5 mM GSH for 2 h prior exposure to compounds and analysis of ROS generation.

### Cell apoptosis analysis

SGC-7901, BGC-823 and KATO III cells were plated on 60-mm dishes for 12 h, and then treated with AF (2, 3 or 4 μM) for 24 h. Cells were then harvested, washed twice with ice-cold PBS, and evaluated for apoptosis by double staining with FITC conjugated Annexin V and Propidium Iodide (PI) in binding buffer for 30 min using a FACSCalibur flow cytometer (BD Biosciences, CA).

### Western blot analysis

Cells or tumor tissues were homogenized in protein lysate buffer, and debris was removed by centrifugation at 12,000 rpm for 10 min at 4°C. The protein concentrations in all samples were determined by using the Bradford protein assay kit (Bio-Rad, Hercules, CA). After addition of sample loading buffer, protein samples were electrophoresed and then transferred to poly-vinylidene difluoride transfer membranes. The blots were blocked for 2 h at room temperature with fresh 5% nonfat milk in TBST and then incubated with specific primary antibody in TBST overnight at 4°C. Following three washes with TBST, the blots were incubated with horseradish peroxidase-conjugated secondary antibodies for 1 h, and the immunoreactive bands were visualized by using ECL kit (Bio-Rad, Hercules, CA). The density of the immunoreactive bands was analyzed using Image J computer software (National Institute of Health, MD).

### Determination of caspase-3/9 activity

Caspase-3/9 activity in cell lysates was determined using a Caspase-3/9 activity kit (Beyotime Institute of Biotechnology, Nantong, China) according to the manufacturer's protocol. The caspase-3/9 activity was normalized by the protein concentration of the corresponding cell lysate and expressed as percentage of treated cells to that of control.

### Transient transfection of small interfering RNA (siRNA)

The sequences for the CHOP siRNA construct were described previously [19]. SGC-7901 cells (3 × 10^5^/well) were seeded into 6-well plates and cultured for 24 h, and then were transfected with siRNA duplexes against human CHOP (100 nM) or control siRNA by lipofectamine 2000 (Invitrogen) according to manufacturer's protocol. Forty-eight hours posttransduction, the cells were washed with complete media and plated with or without AF for 24 hours for assessing apoptosis.

### Evaluation of mitochondrial membrane potential (Δ*ψ*_m_) and Cytochrome C release

The effects of AF on the cell mitochondrial membrane potential (Δ*ψ*_m_) were examined by fluorescence microscope using JC-1 (Beyotime Biotech, Nantong, China) as specific probe. Cells were treated with AF for 14 h and stained with JC-1 in a humidified atmosphere of 5% CO_2_ at 37°C for 30 minutes. Images acquired from monomer and aggregate were merged and viewed under the Nikon fluorescence microscope (40X amplification, Nikon, Japan). Evaluation of the sub-cellular localization of cytochrome C was done by using fluorescence imaging of cells double-labeled with MitoTracker Green (Molecular Probes) and cytochrome C antibody. After treatment, cells were incubated with 100 nM MitoTracker Green, fixed with 3% paraformaldehyde, permeabilized with 0.02% Triton X and blocked with 5% BSA, followed by treatment with primary rabbit polyclonal cytochrome C antibody for 2 h at room temperature and Cy2-conjugated goat anti-rabbit antibody for 1 h. Cellular images were acquired by using a fluorescence microscope (40X amplification, Nikon, Japan).

### *In vivo* antitumor study

All animal experiments were complied with the Wenzhou Medical University's Policy on the Care and Use of Laboratory Animals. Protocols for animal studies were approved by the Wenzhou Medical College Animal Policy and Welfare Committee (Approved documents: 2012/APWC/0216). Five-week-old athymic BALB/cA nu/nu female mice (18–22 g) purchased from Vital River Laboratories (Beijing, China) were used for *in vivo* experiments. Animals were housed at a constant room temperature with a 12 h : 12 h light/dark cycle and fed a standard rodent diet and water. SGC-7901 cells were harvested and injected subcutaneously into the right flank (5 × 10^6^ cells in 100 μL of PBS). When tumors reach a volume of 40–50 mm^3^, mice were treated by intraperitoneal (i.p.) injection of 4 mg/kg PL once per day, or by i.p. injection of 2 mg/kg AF once per day, or with a combination of PL and AF according to the same schedules. The tumor volumes were determined by measuring length (l) and width (w) and calculating volume (V = 0.5 × l × w^2^) at the indicated time points. At the end of treatment, the animals were sacrificed, and the tumors were removed and weighed for use in proteins expression studies.

### Statistical analysis

All experiments were assayed in triplicate (*n* = 3). Data are expressed as means ± SEM. All statistical analyses were performed using GraphPad Pro. Prism 5.0 (GraphPad, SanDiego, CA). Student's *t*-test and two-way ANOVA were employed to analyze the differences between sets of data. A *p* value < 0.05 was considered statistically significant.
